# Weight Management Quality Improvement Project in Tower Hamlets Community Learning Disability Service

**DOI:** 10.1192/bjo.2024.360

**Published:** 2024-08-01

**Authors:** Lucy Dundas, Irem Deniz, Genevieve Hirsz, David Prior, Nicole Eady

**Affiliations:** East London Foundation Trust, London, United Kingdom

## Abstract

**Aims:**

The project's aim is to record up-to-date BMI readings of 70% or more of our service users by September 2024. We have identified barriers limiting current data collection, such as challenges weighing wheelchair bound clients or limited availability of weighing scales, and will action our change idea methods to reach our target in this time period.

Significant health inequalities have been identified in the learning disability population, with men and women in our cohort dying 23 years and 27 years younger respectively compared with the general population. Furthermore, people with learning disabilities are at increased risk of being overweight or obese compared with other cohorts, which itself leads to a range of health and social complications. A recent audit of our psychiatry caseload revealed the need to improve weight monitoring and subsequent management for our service users, to help reduce health inequalities identified.

**Methods:**

We have weekly project meetings with our MDT including psychiatrists, dietetics, occupational therapists, nurses and psychologists. We have arranged stakeholder involvement by inviting service users to these weekly meetings to contribute their own ideas to the project, and have organised focus groups for service users, carers and staff. We intend to generate change ideas by using quality improvement methodology to identify primary and secondary drivers. One of these already incorporated into the project is a machine in our waiting room monitoring our clients’ weight, height and blood pressure. Having identified obstacles in our service users obtaining their weight, we have successfully bid for funding for one of these machines.

**Results:**

We will use Plan Do Study Act (PDSA) cycles to evaluate the effectiveness of our change ideas. Convenient sampling of our psychiatry caseload showed only 26.7% of 71 service users have an updated weight and BMI, and identified that we don't have a robust process for monitoring patients' weights (total project caseload is 1264).

**Conclusion:**

During the development of this project, we identified a variety of approaches to improve health outcomes for our service users including educating staff on incorporating weight monitoring into consultations and how to manage the results. This project comprises one part of East London Foundation Trust's overall Triple Aim: to improve population health; improve the quality and improve value for the system. Going forward, our intention is to incorporate weight management into our routine reviews and ensure staff are educated in the importance of regular weight monitoring, the health benefits and how to refer.

